# Costs of illness in amyotrophic lateral sclerosis (ALS): a cross-sectional survey in Germany

**DOI:** 10.1186/s13023-020-01413-9

**Published:** 2020-06-12

**Authors:** Erik Schönfelder, Alma Osmanovic, Lars Hendrik Müschen, Susanne Petri, Olivia Schreiber-Katz

**Affiliations:** Department of Neurology, Hannover Medical School, OE 7210, Carl-Neuberg-Strasse 1, 30625 Hannover, Germany

**Keywords:** Amyotrophic lateral sclerosis (ALS), Cost of illness (COI), Health care burden, Disease cost, King’s staging system, Health-related quality of life (HRQoL), Socioeconomic burden

## Abstract

**Background:**

Amyotrophic lateral sclerosis (ALS) is a fatal neurodegenerative disorder. Consequently, patients undergo a multidisciplinary treatment that often requires intensive use of medical resources. This study provides an estimate on the cost of illness depending on the clinical severity while also analysing the patients’ health-related quality of life.

**Methods:**

Primary data from patients and caregivers was collected through a standardised questionnaire. Direct medical, direct non-medical and indirect costs were calculated using the latest German health economic guidelines. Patients were divided into five groups according to the King’s staging system. Health-related quality of life was assessed using EuroQoL Group EQ-5D-5L™ questionnaire. Influencing factors on both total cost and quality of life were examined.

**Results:**

The mean annual total cost of illness was 78,256€ per patient while the lifetime cost per patient was estimated at 246,184€. The prevalence based total burden yearly therefore was 519,776,352€ in Germany. Nearly half of the costs were attributable to informal care. With increase of the clinical severity stage, costs rose and quality of life decreased. The score of the revised Amyotrophic Laterals Sclerosis Functional Rating Scale was identified as one major influencing factor on total costs, while subjective impairment in daily activities and classification into a care level as opposed to having no care level influenced patients’ quality of life.

**Conclusion:**

It is essential to understand the socioeconomic burden of a disease. These data can be used to improve patient care standards and quality of life while also serving as a basis for cost-benefit analyses during the approval process of new treatments.

## Background

Amyotrophic lateral sclerosis (ALS) is a progressive neurodegenerative disease that is characterised by upper and lower motor neuron loss. It leads to atrophy and weakness of voluntary muscles, bulbar symptoms like dysarthria and dysphagia and, consequently, to complete paralysis and death after an average of three to five years due to respiratory failure [1, 2]. Its incidence in Germany is estimated at 3.1/100,000, its prevalence at 8/100,000 [3]. The peak age of onset is from 58 to 63 years [4]; and while there is a small proportion of familial ALS cases, the majority (90%) is sporadic [5]. At present, ALS has no known cure; approved drugs only result in a short delay in disease progression [6, 7]. The disease typically worsens over a short period of time and causes severe impairments in the lives of patients and their families. In addition to neuroprotective medication, attempts of pharmacological and supportive alleviation of symptoms and complications are the only other therapeutic option [8, 9]. Regarding these symptomatic measures, for example respiratory support (non-invasive ventilation) and implementation of specialised multidisciplinary ALS clinics have been proven to prolong patient’s life span and, on the other hand, improve their individual quality of life [10, 11]. The increased need for multidisciplinary medical care and management from professionals and relatives leads to considerable social and economic costs [12–15].

Current cost evaluations of larger ALS patient cohorts in Germany are still missing. Therefore, this study aimed to give an estimate of these costs from a societal perspective and to relate them to the patients’ health-related quality of life (HRQoL). These data may contribute to the development of improvements in the patient’s care and quality of life as well as to the acceleration of approval of novel future therapies.

## Methods

### Patient recruitment

Data collection for this explorative cross-sectional study took place between July 2018 and February 2019. Adult patients diagnosed with clinically possible, probable (incl. laboratory supported) and definite ALS, following the revised El Escorial criteria [16], were enrolled into this monocentric study if they had been treated during the past two years at Hannover Medical School (MHH), Department of Neurology. Patients with other motor neuron diseases were also enrolled but not analysed within this paper. Ethical approval for this study was obtained from the institutional ethics board of MHH.

### Data collection

After having given written consent, patients together with their main caregiving relatives answered a standardised questionnaire by hand. The questionnaire was self-designed as described before [17, 18] and pretested in patients with another severe motor neuron disease (spinal muscular atrophy). Besides patients’ demographics and their disease course, the questionnaire recorded individual disease severity by the impairment displayed in daily activities and the details on the current care and medical treatments as the basis of cost estimation. The revised Amyotrophic Lateral Sclerosis Functional Rating Scale (ALSFRS-R), a self-reported outcome that consists of four subdomains, was used to assess disease severity. The ALSFRS-R indicates the level of impairment of distinct motor functions (bulbar, fine and gross motor and respiratory function; maximum 48 points which means no impairment and minimum 0 points which means total dependence [19]).

For further analysis, the patients were subdivided into five groups according to the King’s clinical staging system for ALS [20], which was derived from the ALSFRS-R score as suggested before [21]. This system categorises the progressive clinical involvement of anatomical regions throughout the course of the disease. Of the five King’s stages, each one is associated with the number of regions involved and classified as follows: stage 1 one functional region involved; stage 2 two regions; stage 3 three regions; stage 4A corresponds to nutritional failure (gastrostomy); stage 4B to respiratory failure and the indication of assisted ventilation; stage 5 corresponds to death. Health-related quality of life (HRQoL) and health status were measured by the EuroQol Group EQ-5D-5L™ in the five dimensions Mobility, Self-Care, Usual Activities, Pain/Discomfort and Anxiety/Depression [22]. These dimensions are rated in five levels from no problems (= level 1) to extreme problems (= level 5). The scores are weighted based on preference of discrete combinations of health states (e.g. 11111 or 11112 and so on). Health states were translated into an EQ-5D-5L™ index value by use of the German value set as recommended (1 = best state, 0 = worst state, further referred to as index value) [23]. Additionally, the patient’s self-rated health on a visual analogue scale (VAS; 100 = the best imaginable health and 0 = worst imaginable health) was recorded (further referred to as EQ-VAS score).

### Cost estimation

The total cost of illness (COI) can be subdivided in different cost categories, namely direct and indirect costs. Direct costs require direct payments from patients, insurances or third parties. They can be differentiated into direct medical (e.g. drugs, supportive medical devices, doctors’ consultations, hospital treatments, surgery, rehabilitations, further therapies like physio-, ergo- and speech therapy and formal care) and non-medical costs (e.g. travel expenses, investments in constructional alterations, legal fees and informal care – which means care provided by non-professional caregivers, in the majority family members). We used a micro-costing method to assess costs from a societal perspective [24].

The utilisation of medical and non-medical resources was assessed retrospectively within different recall periods in order to reduce recall bias. The current status was assessed for supportive devices and formal care (for partial and complete inpatient care three or twelve months, respectively). According drugs, the individual utilisation within the past week was reported, for informal care within the past two weeks and within the past month for further therapies and psychological support. Outpatient physician and hospital consultations were assessed with respect to the past three months, while hospitalisations and sleep laboratory were assessed for the past six months, vaccination and rehabilitation for the past twelve months. Moreover, we surveyed the individual utilisation of surgery, legal support, constructional alterations and travel expenses corresponding to the reason for travelling ever since disease manifestation. The utilisation of direct medical and non-medical resources (see Additional file 1) was valued monetarily by unit prices based on the latest health economic recommendations for Germany [25–27]. Moreover, patients were asked about their current classification into a care level according to the German health care system (and acquired services). Here, higher levels indicate a greater loss of autonomy and a need for more individual support (care level 1 = low impairment of individual autonomy, care level 5 = most severe impairment of individual autonomy with special demands for nursing care) [28]. For the calculation of informal care costs, we replaced the time of care provided by informal caregivers by the statutory minimum wage for the caring sector in Germany and thus estimated the costs that would have risen if the care had been provided by professional caregivers instead [25].

In contrast, indirect costs result from absent times from work and invalidity of the affected person (and probably his caring relative) and represent the loss of productivity. Accordingly, we used the human capital approach and calculated monetary losses due to reduction of working time, absent days and early retirement based on patient reported salary levels [27]. For a more detailed methodology of cost estimation see Additional file 2.

All costs were extrapolated to one year while we assumed a stable status over this time period. Costs were shown in Euros (€) for the year 2018 (enquiry period).

### Statistical analysis

Statistical analysis was completed using IBM SPSS 25®. Demographic data was determined using frequency tables, and normal Gaussian distribution was tested using Kolmogorov-Smirnov-test. Average differences between subgroups were tested using students T-test and correlation was tested using Spearman rank correlation coefficient. Because of the explorative character of this study, we did not adjust for multiple testing. All statistical results have to be understood as hypothesis generating and not as confirmatory. Regression analysis was done by analysing possible influencing factors in a simple linear regression model. If a variable turned out to be significant (two-sided *p*-value of < 0.05) we entered it into the final multiple linear regression models.

## Results

### Patient characterisation

156 patients were included in the analysis (response rate 37.6%: 187/497; the majority of patients were from Northern Germany, Lower Saxony 66.7%). The patients’ age range was from 27 to 86 with the median being 65 years (Table 1). 60.3% were male which is in line with findings that ALS is more common in men [29]. The median disease duration from symptom onset was two years. Patients showed a different distribution of ALS phenotypes. 35.3% reported a bulbar onset whereas 34% reported a lower limb onset which approximates previous reports [3]. The median ALSFRS-R score was 30, ranging from 1 to 48. 110 patients were classified into care levels (75.3%) where the majority of patients had a moderate to most severe loss of autonomy (care levels 3–5) and were impaired in daily activities (95.5%) or even needed the permanent attendance of another (care-giving) person 24 hours (h) a day (39.1%). Most patients (85.7%) were married or lived with a partner who was the primary caregiver (86%, see Additional file 1). 17.3% had private health insurance, which is slightly more than the general proportion within the German population [30]. At the time-point of the survey, only 13.8% of patients were still working in contrast to 47.1% of caregivers (Table 1).

**Table 1:** Demographics

	Percent or median (IQR)	Absolute number of patients
Age, y	65 (17)	
Sex, female	39.7	62
BMI	24.03 (5.12)	
Type of health insurance (*n* = 150), statutory	82.7	124
Symptom onset, y	62 (17.8)	
Disease duration from symptom onset, y	2 (3)	
Inherited ALS	3.8	6
ALSFRS-R score (max. 48)	30 (16)	
King’s staging		
1	12.8	20
2	26.3	41
3	28.2	44
4A	10.3	16
4B	22.4	35
Level of care (*n* = 146)		
none	24.7	36
1	2.1	3
2	11.6	17
3	28.1	41
4	17.1	25
5	16.4	24
Self-rated impairment in daily activities	95.5	149
Permanent attendance of a caregiver necessary	39.1	61
Main caregiver (*n* = 136)		
Partner	86	117
Children	7.4	10
Others	6.6	9
Employment situation of the main caregiver (*n* = 119)		
Working caregiver	47.1	56
Main caregiver stopped working because of patient’s ALS	5	6
Change of weekly working time because of patient’s ALS	14.3	17
Job change because of patient’s ALS	2.5	3
Drop in salary because of patient’s ALS	10.1	12
Housing situation (*n* = 152)		
Family	87.5	133
Alone	10.5	16
Assisted living/foster home	2	3
Employment (*n* = 145)		
Employment no longer possible	26.2	38
Working	13.8	20
Retired, unemployed, homemaker	60	87
Reasons for unemployability (*n* = 34)		
Retired because of ALS	61.8	21
Unable to work	5.9	2
Unknown	32.4	11
EQ-VAS score (max. 100)	40 (35)	
EQ-5D-5L™ index value (max. 1)	0.585 (0.623)	

This table shows the most important patient characteristics, their disease stage (King’s staging), and the impairment in their autonomy and working lives. The professional activity assessment of patients and of their main caregivers served as basis for the calculation of indirect costs

*Abbreviations*: *IQR* interquartile range, *y* years, *BMI* body mass index, *n* number, *ALS* amyotrophic lateral sclerosis, *ALSFRS-R* Revised Amyotrophic Lateral Sclerosis Functional Rating Scale, *EQ-VAS score* Self-rated health on the visual analogue scale of EuroQol Group EQ-5D-5L^TM^ instrument. health on a visual analogue scale (0–100)

### Utilisation of medical resources

**Fig. 1:**
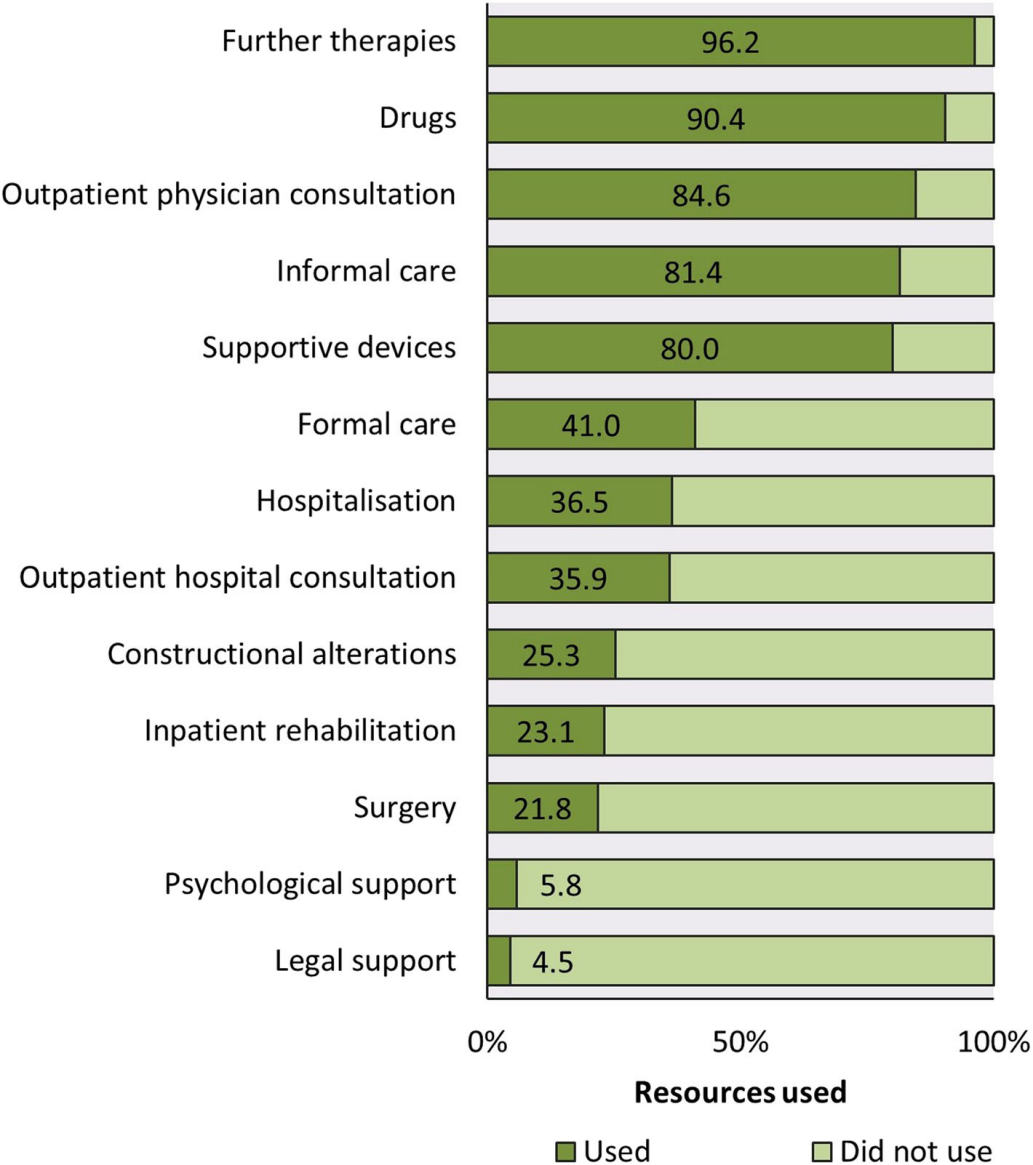
Utilisation of medical resources. Figure 1 shows the proportion of the use of different medical resources. Further details are shown in Additional file 1

The assessment of the use of medical (and non-medical) resources served as calculation basis for direct medical and non-medical costs (Fig. 1). It reflected the present patients’ care and their access according to current standards. The detailed data is shown in Additional file 1.

Nearly all patients (96.2%) were treated with further therapies. The most common was physiotherapy (82.7%, mean visits per year, 88), speech therapy (62.2%, mean visits per year, 48) and ergotherapy (59.6%, mean visits per year, 45). 84.6% had consulted an outpatient physician during the last three months (mean visits per year: 13) with the most commonly frequented one being a neurologist (67.3%). 80% made use of supportive devices. On average, every patient used seven different devices, most commonly mobility devices such as wheelchairs and walking frames (67.9%). Inpatient rehabilitation (23.1%) and psychological support (5.8%) were used less frequently. There was a strong contrast between the frequency of use of formal (41.0%) and informal (81.4%) care. Daily, the patients needed 1.4 h of formal care when compared to 7.6 h of informal care. The most common form of formal care was domestic aid (31.4%) (Fig. 1).

### Cost of illness (COI)

**Fig. 2:**
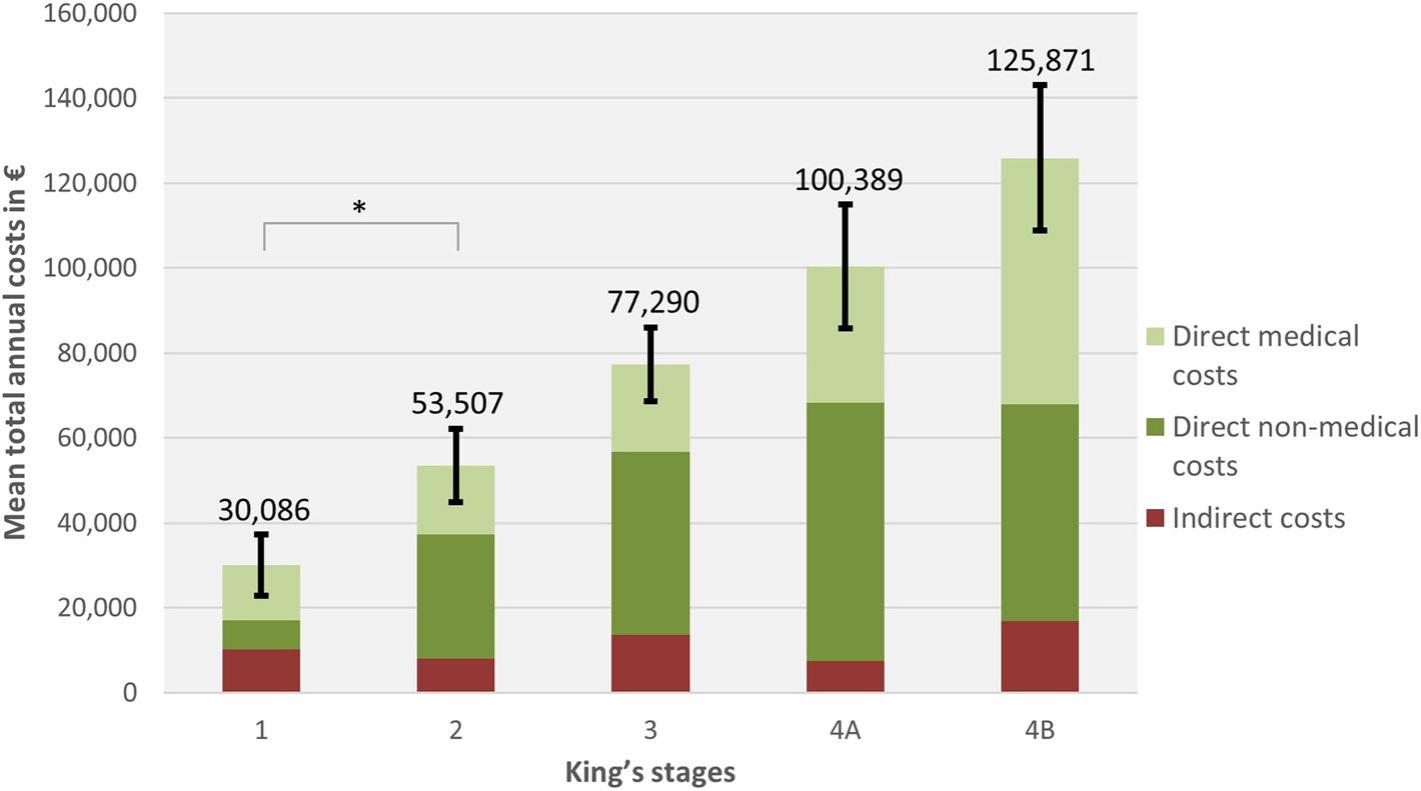
Annual cost of illness (COI) according to King’s staging. Standard errors presented refer to the total annual costs. Significant positive correlations between King’s staging and total costs (r_s_ = 0.482, *p* < 0.001, *n* = 156), between King’s staging and direct medical costs (r_s_ = 0.361, p < 0.001, n = 156) and between King’s staging and direct non-medical costs (r_s_ = 0.450, p < 0.001, n = 156). No significant correlations were found between King’s staging and indirect costs (r_s_ = 0.102, *p* = 0.205, n = 156). Abbreviations: € = Euro. * = *p* < 0.05

The average COI was estimated to be 78,256€ per patient yearly (Table 2). Direct medical costs constituted 35.9%, direct non-medical costs 49.1% and indirect costs 15% of total COI. The main cost driving factors were informal care (46.2%), indirect costs (15%) and formal care (11.4%). Total COI increased with every clinical severity stage (Fig. 2). There was a significant positive correlation between King’s staging and total costs (r_s_ = 0.482, *p* < 0.001), direct medical costs (r_s_ = 0.361, p < 0.001) and direct non-medical costs (r_s_ = 0.450, p < 0.001). However, there was no significant correlation between King’s staging and indirect costs (r_s_ = 0.102, *p* = 0.205).

**Table 2:** Cost of illness (COI)

	Mean annual costs in € (95% CI)	Ratio of total COI (percent)
**Direct medical costs**	**28,087 (20,911-35,263)**	**35.9**
Formal care	8888 (2601-15,174)	11.4
Further therapies	7629 (6610-8649)	9.7
Hospitalisation	4568 (2991-6145)	5.8
Supportive devices	2785 (2032-3538)	3.6
Drugs	2190 (1971 − 2409)	2.8
Inpatient rehabilitation	885 (607–1162)	1.1
Outpatient physician consultations	612 (515–710)	0.8
Surgery	189 (114–263)	0.2
Outpatient hospital consultations	180 (140–220)	0.2
Psychological support	161 (29–293)	0.2
**Direct non-medical costs**	**38,412 (31,695-45,130)**	**49.1**
Informal care	36,152 (29,621-42,683)	46.2
Constructional alterations	1871 (1123-2618)	2.4
Travel expenses	353 (270–436)	0.5
Legal support	9 (1–18)	0.0
Other costs	27 (24–31)	0.0
**Indirect costs**	**11,757 (8232-15,282)**	**15.0**
**Total COI**	**78,256 (66,583-89,929)**	**100.0**

Incurred costs per ALS patient and year in the different cost categories from a societal perspective. Other costs consisted of e.g. ALS-related fitness centre membership and others. Due to rounding, percentages do not add up exactly

*Abbreviations*: *€* Euro, *CI* confidence interval

Influencing factors on total annual COI are shown in Table 3. Total costs increased with the decline of each point of the ALSFRS-R by 2333€ (95% CI [1082€, 3584€], *p* < 0.001). The highest increase of costs was seen in cases where patients required invasive ventilation. In this patient group, costs were substantially higher compared to patients who did not have invasive ventilation (plus 47,803€, 95% CI [5112€, 90,493€], *p* = 0.029). If patients required the permanent attendance of a caregiver, costs rose by 22,178€ (95% CI [2684€, 41,671€], *p* = 0.026) and if they were wheelchair dependent, costs increased by 14,081€ (95% CI [1388€, 26,774€], *p* = 0.030).

**Table 3:** Influencing factors on total COI

Variable	Change in total costs, €	95% CI	*p*-value
ALSFRS-R score (max. 48)	-2333	−3584 to −1082	0.000
Permanent attendance of a caregiver necessary	22,178	2684 to 41,671	0.026
Invasive ventilation	47,803	5112 to 90,493	0.029
Wheelchair use	14,081	1388 to 26,774	0.030
Underweight	14,378	−9564 to 38,320	0.237
Classified into a care level	− 6491	−27,461 to 14,479	0.541
Feeding tube use	7617	−17,849 to 33,084	0.555
Currently working	− 6150	−28,304 to 16,005	0.584
n	127		

This multiple linear regression model showed significant influence of the ALSFRS-R score, wheelchair use, invasive ventilation, and the necessity of permanent attendance of another caregiver on total costs. The model was adjusted for statistical outliers

*Abbreviations*: *COI* cost of illness, *€* Euro, *CI* confidence interval, *ALSFRS-R* Revised Amyotrophic Lateral Sclerosis Functional Rating Scale, *n* number

Roche et al. described a mean disease duration of 42.3 months until death and standardised time periods from disease onset to every clinical King’s stage [20]. Respectively, patients reach King’s stage 2 after 17.7 months, stage 3 after 23.3 months, stage 4A after 27.7 months and stage 4B after 30.3 months. By multiplying the average time expected to be in one stage with the average costs estimated for that stage and adding the results for all stages, the lifetime costs of a patient suffering from ALS from symptom onset to death were estimated at a total of 246,184€. Moreover, the prevalence-based total costs in Germany can be added up to 519,776,352€ per year (prevalence of ALS 8/100,000 [3] in a population of 83,019,200 in 2018 [31]).

#### Health-related quality of life (HRQoL)

**Fig. 3:**
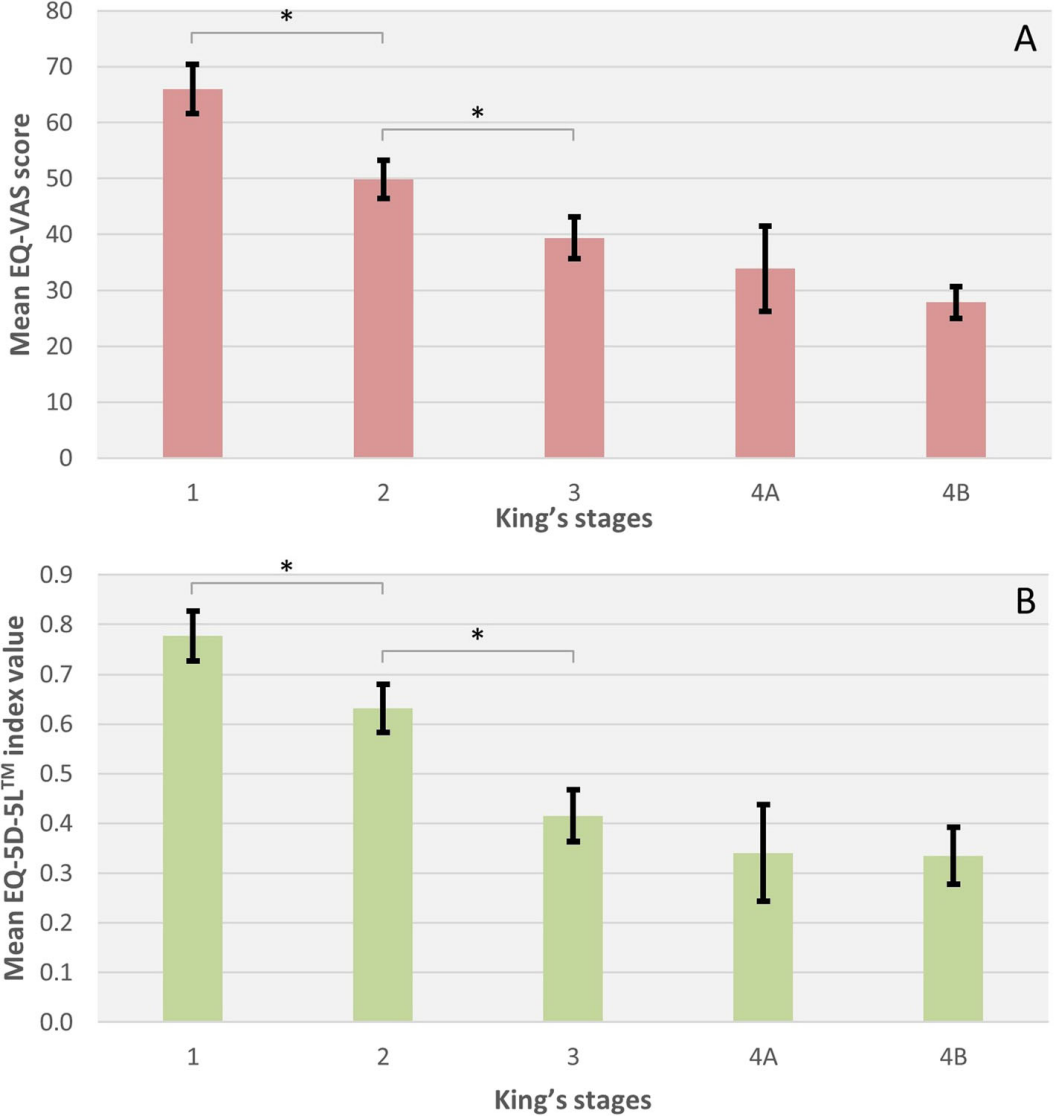
Health-related quality of life (HRQoL) according to King’s staging. Mean scores are presented with standard errors. **a** shows mean EQ-VAS scores, **b** shows mean EQ-5D-5L™ index values. Significant negative correlation between King’s staging and EQ-VAS score (r_s_ = − 0.490, p < 0.001, *n* = 141) as well as EQ-5D-5L™ index values (r_s_ = − 0.477, p < 0.001, n = 141). Abbreviations: EQ-VAS score = Self-rated health on the visual analogue scale of EuroQol Group EQ-5D-5L^TM^ instrument. health on a visual analogue scale (0–100). * = p < 0.05

**Fig. 4:**
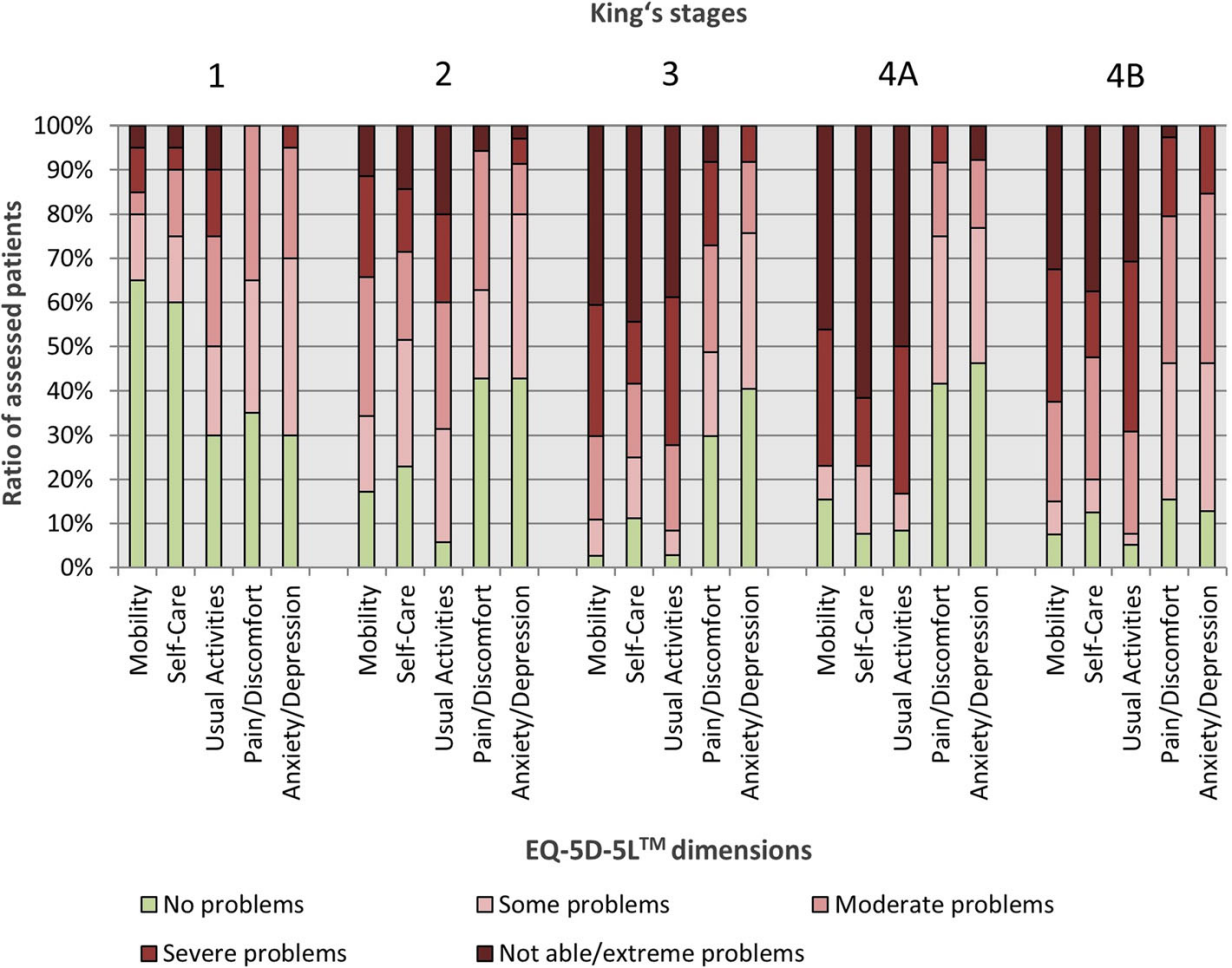
Impairments in the five domains of EQ-5D-5L™ according to King’s staging. Impairments in the five dimensions increased with disease severity, the domains Mobility, Self-Care and Usual Activities were impaired to a higher degree and remained in focus within the disease course

HRQoL was measured by EQ-5D-5L™. The median EQ-VAS score, which reflects the self-rated current health on a visual analogue scale from 0 to 100, was 40. The EQ-5D-5L™ index value, which is derived from five dimensions of HRQoL (Mobility, Self-Care, Usual Activities, Pain/Discomfort, Anxiety/Depression), showed a median value of 0.59 (Table 1). HRQoL was inversely correlated to disease progression. There was a significant negative correlation between King’s staging and EQ-VAS score (r_s_ = − 0.490, *p* < 0.0005, *n* = 141, Fig. 3a) and King’s staging and index value (r_s_ = − 0.477, p < 0.0005, n = 141, Fig. 3b). Patients already stated impairments in all five domains in King’s stage 1, but only to a lower degree (mainly no or some to moderate problems). In more advanced disease stages, severe to extreme problems or even losing the ability in the domains Mobility, Self-Care and Usual Activities overwhelmingly dominated, accentuated between King’s stage 2 to stages 3, 4A and 4B. The dimensions Pain/Discomfort and Anxiety/Depression were only impaired to a low to moderate extent even in stage 4B (Fig. 4).

Table 4 shows the influencing factors on the HRQoL (EQ-VAS score and index value). Patients, who stated to have general impairment in daily activities, self-rated their HRQoL to be 24.7 points lower on a visual analogue scale than patients who did not feel impaired (95% CI [− 41.7; − 7.7], *p* = 0.005) and showed a decrease of 0.15 points of the index value (95% CI [− 0.28; − 0.02], *p* = 0.023). Decreases of 14 points in the EQ-VAS score (95% CI [− 22.3; − 5.6], *p* = 0.001) and 0.13 in the index value (95% CI [− 0.22; − 0.04], p = 0.005) were observed, if a patient was classified into a care level. Moreover, wheelchair dependency (− 8.8 points in the EQ-VAS score (95% CI [− 16; − 4], *p* = 0.030) and − 0.17 in the index value (95% CI [− 0.22; − 0.12]. *p* < 0.0005)) and also the need of permanent attendance of a caregiver (− 8.3 in the EQ-VAS score (95% [CI -16.1; − 0.5], *p* = 0.037) and − 0.25 in the index value (95% CI [− 0.33; − 0.16], p < 0.0005)) showed to be main influencers of HRQoL. Additionally, age showed to be an influencing factor on EQ-VAS score (− 0.3, 95% CI [− 0.6; 0], *p* = 0.034; Table 4).

**Table 4:** Influencing factors on HRQoL

**A Influencing factors on EQ-VAS score**			
Variable	Change in EQ-VAS score	95% CI	*p*-value
Classified into a care level	−14	−22.3 to −5.6	0.001
Impaired in daily activities	−24.7	−41.7 to −7.7	0.005
Wheelchair use	−8.8	−16 to −4	0.030
Age	−0.3	−0.6 to 0	0.034
Permanent attendance of a caregiver necessary	−8.3	−16.1 to −0.5	0.037
n	131		
**B Influencing factors on EQ-5D-5L™ index value**			
Variable	Change in EQ-index score	95% CI	*p*-value
Permanent attendance of a caregiver necessary	−0.25	−0.33 to −0.16	0.000
Wheelchair use	−0.17	−0.22 to −0.12	0.000
Classified into a care level	−0.13	−0.22 to −0.04	0.005
Impaired in daily activities	− 0.15	−0.28 to −0.02	0.023
Invasive ventilation	−0.13	−0.28 to 0.03	0.103
Currently working	−0.02	−0.12 to 0.08	0.692
n	122		

This multiple linear regression model showed significant influence by mainly of loss of autonomy (classification into a care level, impairment in daily activities, wheelchair use and need of permanent attendance of a caregiver) on HRQoL. The model was adjusted for statistical outliers

*Abbreviations*: *HRQoL* health related quality of life, *€* Euro, *EQ-VAS score* Self-rated health on the visual analogue scale of EuroQol Group EQ-5D-5L^TM^ instrument. health on a visual analogue scale (0–100), *CI* confidence interval, *n* number

## Discussion

In this study, we analysed the COI of ALS in Germany related to the already well established and disease-specific King’s staging system. As far as we know, our study investigated the greatest patient cohort in Germany by now and presents an estimate for the COI of ALS, which is based on current health-economic recommendations. Our estimates give an assessment basis for further cost effectiveness studies and payer negotiations. As far as we know, cost effectiveness studies are rare in the field of ALS as it is a rare disease and treatments are limited up to date [32–35]. Nevertheless, the necessity of these studies is currently increasing due to the investigation of different new treatment approaches [36–38]. However, the conduction of cost analyses in ALS is difficult, since the health insurance companies in Germany are not transparent about their expenses [25]. Real-life expenditures of the German health system would provide a fruitful comparison; however, these are not accessible to the greater public. Our estimates for mean annual costs varied from 30,086€ (King’s stage 1) to 125,871€ (King’s stage 4B). This demonstrates a high socioeconomic burden even in early stages of the disease. The mean annual total COI per patient was 78,256€, which is 17-times higher than average costs per capita in Germany (4544€) [39]. The highest cost components were informal care costs (46.2%) and direct medical costs (35.9%, formal care 11.4% of total COI), while indirect costs constituted 15% of total COI. Patients’ family members are the main contributors to informal care in Germany [40, 41]. In our cohort, in 94.1% of the cases the main caring person was a family member. This plays a significant role not only in terms of the psychological burden but also from a socioeconomic perspective. Caregivers of patients with chronic diseases are reported to have poorer mental and physical health compared to non-caregivers [42]. This may result in even higher ALS-related costs caused by an additional need for treatment and loss of productivity on the caregivers’ side.

Compared to previous studies in neuromuscular disorders (NMD), we estimated higher informal care costs (46.2% vs. 27% in Duchenne muscular dystrophy (DMD), 20% in Becker muscular dystrophy (BMD), 29% in spinal muscular atrophy (SMA) and 33.6% in Charcot-Marie-Tooth neuropathies (CMT)) [17, 18, 43]. Among different NMD, associated costs of ALS were the highest regarding both, informal care costs and total COI, which also corresponds to similar studies in the past [12, 13, 15]. In contrast, indirect costs were much higher in DMD, BMD, SMA and CMT. While in our cohort direct medical and non-medical costs were correlated to increase in clinical severity, indirect costs did not. A possible explanation for these differences may involve our cohort’s demographics: as the median age was 65 and 60% were already retired at the time of the survey for non-disease related reasons, productivity loss because of ALS was low and independent from clinical severity throughout our cohort. Our study did not consider indirect costs due to premature mortality. However, these costs may probably be negligible due to the late disease onset and as the majority of patients was already retired.

We showed that COI was higher and HRQoL was lower in more advanced disease stages. The influencing factors with the highest impact on COI were the ALSFRS-R score and invasive ventilation. On the other hand, HRQoL was significantly influenced by individual impairment in daily activities and classification into a care level. Common influencing factors on both, COI and HRQoL, were wheelchair dependency and the need of presence of an additional caregiver 24 h per day. This shows that progressive loss of autonomy and increasing functional impairment throughout disease progression is the main cause of cost increase while additionally determining a reduced self-rated quality of life.

Our analysis of HRQoL showed a difference between EQ-VAS score and index value; patients reported their health state on the EQ-VAS score to be worse than what the index value showed (Median of 40/100 versus 0.59/1). Those results, however, are not surprising as the EQ-VAS score is known to be also influenced by factors such as perceived control, education, ethnicity, smoking and age [44], while the latter proved to be an influencing factor on EQ-VAS in our linear regression model as well.

ALS patients experience major impairments in their everyday lives and need a lot of support to maintain at least a residual independence. This is underlined by our patient cohort, in which 80% needed supportive devices, on average seven different devices per patient. In contrast, supportive devices only constituted 3.6% of total COI. While this figure may seem low, a study from the United States, which had access to “true costs” from insurance companies, confirmed this result [12]. Concerning the supply of supportive devices, we made the following substantial observations: 20.2% of patients who stated to have impairment of their mobility according to the EQ-5D-5L™ did not use any mobility device. Moreover, 28.9% who were impaired in self-care did not use special devices and 75.6% who were impaired in daily activities, also did not use support in this regard. While depression is known to occur in 22.8% of patients with ALS [45], only 5.8% made use of psychological support. Interestingly, of 52 patients who stated a need for improvement in disease related support, 15 (28.8%) wished for support in the application for supportive medical devices and 12 (23%) for better and easier accessible psychological support. Similarly, regarding the ALSFRS-R results, of 88 patients who experienced dyspnoea, 52 (59.1%) did not use any breathing assistance. These findings strongly suggest a supply gap in these areas and maybe especially in the medical supply with more costly devices like ventilators (and the associated care that is needed with them). Better access to support and, perhaps, even costlier devices to maintain a higher grade of autonomy would raise the ratio of costs for supportive devices. Nevertheless, a supply in agreement with standards of care is necessary and not only increases the individual quality of life but also may lower other cost factors like informal care costs or indirect costs.

Our estimate of a total annual COI of 78,256€ in Germany is higher than previously assumed by Schepelmann et al. in 2010 (36,380€) [15]. Compared to previous studies in other countries, e.g. Spain (36,194€) [13], US (63,693$) [12] and Korea (90,000$) [14], our estimates are on the higher side of estimated costs. However, direct comparisons must be done with caution due to respective healthcare systems and different approaches in cost estimation. Regardless, previous studies also described disease severity, need for a caregiver, wheelchair dependency and invasive ventilation as main cost influencing factors [12, 15], which further validates our model.

Compared to other NMD such as Myasthenia gravis (14,950€) [15], CMT (17,427€) [43], facioscapulohumeral muscular dystrophy (26,240€) [15] and BMD (39,060€) [17] total COI of ALS is notably higher, similar to those of DMD (78,913€) [17] and SMA (70,566€) [18]. Due to a higher prevalence of ALS, the Germany-wide annual cost was 519,776,352€ which is more than three times higher than the estimates for DMD and nearly five times higher than the estimates for SMA.

To estimate informal care costs, we used a replacement cost approach, which is known to result in overestimation due to informal care being considered less efficient than formal [24]. On the other hand, the associated psychological and physical burden of the caregivers who provided informal care was not reflected and may result in substantial underestimation of costs. Since our questionnaire was rather detailed with more than 120 questions, a selection bias towards more motivated patients might be likely. Moreover, patients were asked to answer questions regarding use of medical resources up to one year in the past, which probably led to recall bias, and further reduced total COI. Another limitation of our study is the monocentric design and coverage mainly of Northern Germany. A multicentre Germany-wide study that employs a greater patient cohort would be desirable to achieve an even more representative result. Thus, further studies of disease burden of ALS that also consider the burden of the caregivers are necessary.

## Conclusion

The results of our descriptive study confirm previous publications and show that costs in ALS increase with disease severity, are much higher than in other NMD and are mainly influenced by patients’ individual autonomy status. Loss of autonomy and disease progression also significantly reduce the self-rated quality of life. Our study underlines that ALS ranks among the most costly neurological diseases and therefore, even though it is a rare disease, contributes to a high socioeconomic burden. Search for novel therapies therefore may not only contribute to a better prognosis and quality of life of patients but also might lower societal costs if the disease progression could be stopped in early disease stages. Nevertheless, further cost analyses and cost effectiveness studies in ALS are mandatory.

Our data further highlight the urgent need of a direct and straightforward access to recommended therapies and supportive devices as well as psychological support for all patients. Formal care should be improved to reduce patient and caregiver burden and thus informal care costs and indirect costs.

## Supplementary information

**Additional file 1.** Further details on the utilisation of medical resources.

**Additional file 2.** Detailed description of cost estimation methodology.

## Data Availability

The datasets used and/or analysed during the current study are available from the corresponding author on reasonable request.

## Abbreviations

ALS: Amyotrophic lateral sclerosis
ALSFRS-R: Revised Amyotrophic Laterals Sclerosis Functional Rating Scale
BMD: Becker muscular dystrophy
CI: Confidence interval
CMT: Charcot-Marie-Tooth disease
COI: Cost of illness
DMD: Duchenne muscular dystrophy
EQ-VAS: Self-rated health on the visual analogue scale of EuroQol Group EQ-5D-5L^TM^ instrument
HRQoL: Health-related quality of life
MHH: Hannover Medical School
NMD: Neuromuscular disorders
SMA: Spinal muscular atrophy

## References

[1] Zarei S, Carr K, Reiley L, Diaz K, Guerra O, Altamirano PF, Pagani W, Lodin D, Orozco G, Chinea A (2015). A comprehensive review of amyotrophic lateral sclerosis. Surg Neurol Int.

[2] Foster LA, Salajegheh MK (2019). Motor neuron disease: pathophysiology, diagnosis, and management. Am J Med.

[3] Rosenbohm A, Peter R, Erhardt S, Lulé D, Rothenbacher D, Ludolph A, Nagel G (2017). Epidemiology of amyotrophic lateral sclerosis in southern Germany. J Neurol.

[4] Kiernan MC, Vucic S, Cheah BC, Turner MR, Eisen A, Hardiman O, Burrell JR, Zoing MC (2011). Amyotrophic lateral sclerosis. Lancet.

[5] Swinnen B, Robberecht W (2014). The phenotypic variability of amyotrophic lateral sclerosis. Nat Rev Neurol.

[6] Miller RG, Mitchell JD, Moore DH (2012). Riluzole for amyotrophic lateral sclerosis (ALS)/motor neuron disease (MND). Cochrane Database Syst Rev.

[7] Abe K, Itoyama Y, Sobue G, Tsuji S, Aoki M, Doyu M, Hamada C, Kondo K, Yoneoka T, Akimoto M, Yoshino H (2014). Confirmatory double-blind, parallel-group, placebo-controlled study of efficacy and safety of edaravone (MCI-186) in amyotrophic lateral sclerosis patients. Amyotroph Lateral Scler Frontotemporal Degener..

[8] Miller RG, Jackson CE, Kasarskis EJ, England JD, Forshew D, Johnston W, Kalra S, Katz JS, Mitsumoto H, Rosenfeld J, Shoesmith C, Strong MJ, Woolley SC (2009). Practice parameter update: the care of the patient with amyotrophic lateral sclerosis: drug, nutritional, and respiratory therapies (an evidence-based review): report of the quality standards Subcommittee of the American Academy of neurology. Neurology..

[9] Hobson EV, McDermott CJ (2016). Supportive and symptomatic management of amyotrophic lateral sclerosis. Nat Rev Neurol.

[10] Bourke SC, Tomlinson M, Williams TL, Bullock RE, Shaw PJ, Gibson GJ (2006). Effects of non-invasive ventilation on survival and quality of life in patients with amyotrophic lateral sclerosis: a randomised controlled trial. Lancet Neurol.

[11] Chiò A, Bottacchi E, Buffa C, Mutani R, Mora G (2006). Positive effects of tertiary centres for amyotrophic lateral sclerosis on outcome and use of hospital facilities. J Neurol Neurosurg Psychiatry.

[12] Larkindale J, Yang W, Hogan PF, Simon CJ, Zhang Y, Jain A, Habeeb-Louks EM, Kennedy A, Cwik VA (2014). Cost of illness for neuromuscular diseases in the United States. Muscle Nerve.

[13] López-Bastida J, Perestelo-Pérez L, Montón-Alvarez F, Serrano-Aguilar P, Alfonso-Sanchez JL (2009). Social economic costs and health-related quality of life in patients with amyotrophic lateral sclerosis in Spain. Amyotroph Lateral Scler.

[14] Oh J, An JW, Oh S, Oh KW, Kim JA, Lee JS, Kim SH (2015). Socioeconomic costs of amyotrophic lateral sclerosis according to staging system. Amyotroph Lateral Scler Frontotemporal Degener..

[15] Schepelmann K, Winter Y, Spottke AE, Claus D, Grothe C, Schröder R, Heuss D, Vielhaber S, Mylius V, Kiefer R, Schrank B, Oertel WH, Dodel R (2010). Socioeconomic burden of amyotrophic lateral sclerosis, myasthenia gravis and facioscapulohumeral muscular dystrophy. J Neurol.

[16] Ludolph A, Drory V, Hardiman O, Nakano I, Ravits J, Robberecht W, Shefner J (2015). A revision of the El Escorial criteria - 2015. Amyotroph Lateral Scler Frontotemporal Degener.

[17] Schreiber-Katz O, Klug C, Thiele S, Schorling E, Zowe J, Reilich P, Nagels KH, Walter MC (2014). Comparative cost of illness analysis and assessment of health care burden of Duchenne and Becker muscular dystrophies in Germany. Orphanet J Rare Dis..

[18] Klug C, Schreiber-Katz O, Thiele S, Schorling E, Zowe J, Reilich P, Walter MC, Nagels KH (2016). Disease burden of spinal muscular atrophy in Germany. Orphanet J Rare Dis.

[19] Cedarbaum JM, Stambler N, Malta E, Fuller C, Hilt D, Thurmond B, Nakanishi A (1999). The ALSFRS-R: a revised ALS functional rating scale that incorporates assessments of respiratory function. BDNF ALS study group (phase III). J Neurol Sci.

[20] Roche JC, Rojas-Garcia R, Scott KM, Scotton W, Ellis CE, Burman R, Wijesekera L, Turner MR, Leigh PN, Shaw CE, Al-Chalabi A (2012). A proposed staging system for amyotrophic lateral sclerosis. Brain..

[21] Balendra R, Jones A, Jivraj N, Knights C, Ellis CM, Burman R, Turner MR, Leigh PN, Shaw CE, Al-Chalabi A (2014). Estimating clinical stage of amyotrophic lateral sclerosis from the ALS functional rating scale. Amyotroph Lateral Scler Frontotemporal Degener..

[22] Herdman M, Gudex C, Lloyd A, Janssen M, Kind P, Parkin D, Bonsel G, Badia X (2011). Development and preliminary testing of the new five-level version of EQ-5D (EQ-5D-5L). Qual Life Res.

[23] Ludwig K (2018). Graf von der Schulenburg JM, Greiner W. German Value Set for the EQ-5D-5L. Pharmacoeconomics..

[24] Krauth C (2010). Methoden der Kostenbestimmung in der gesundheitsökonomischen evaluation [cost estimation methods in health economic evaluation]. Gesundh ökon Qual manag.

[25] Bock JO, Brettschneider C, Seidl H, Bowles D, Holle R, Greiner W, König HH (2015). Standardisierte Bewertungssätze aus gesellschaftlicher Perspektive für die gesundheitsökonomische Evaluation.

[26] Bock JO, Brettschneider C, Seidl H, Bowles D, Holle R, Greiner W, König HH (2015). Ermittlung standardisierter Bewertungssätze aus gesellschaftlicher Perspektive für die gesundheitsökonomische evaluation [calculation of standardised unit costs from a societal perspective for health economic evaluation]. Gesundheitswesen..

[27] Krauth C, Hessel F, Hansmeier T, Wasem J, Seitz R, Schweikert B (2005). Empirische Bewertungssätze in der gesundheitsökonomischen evaluation -- ein Vorschlag der AG Methoden der gesundheitsökonomischen evaluation (AG MEG) [empirical standard costs for health economic evaluation in Germany -- a proposal by the working group methods in health economic evaluation]. Gesundheitswesen..

[28] Bundesgesundheitsministerium. Pflegegrade. 2018. https://www.bundesgesundheitsministerium.de/pflegegrade.html. Accessed 11 December 2019.

[29] Manjaly ZR, Scott KM, Abhinav K, Wijesekera L, Ganesalingam J, Goldstein LH, Janssen A, Dougherty A, Willey E, Stanton BR, Turner MR, Ampong M, Sakel M, Orrell RW, Howard R, Shaw CE, Leigh PN, Al-Chalabi A (2010). The sex ratio in amyotrophic lateral sclerosis: a population based study. Amyotroph Lat Scler.

[30] Bundesgesundheitsministerium. Gesetzliche Krankenversicherung: Mitglieder, mitversicherte Angehörige und Krankenstand, Jahresdurchschnitt 2018. 2019. https://www.bundesgesundheitsministerium.de/fileadmin/Dateien/3_Downloads/Statistiken/GKV/Mitglieder_Versicherte/KM1_JD_2018.pdf. Accessed 24 June 2019.

[31] Statistisches Bundesamt. Bevölkerung auf Grundlage des Zensus 2011 nach Geschlecht und Staatsangehörigkeit im Zeitverlauf. 2019. https://www.destatis.de/DE/Presse/Pressemitteilungen/2019/06/PD19_244_12411.html. Accessed 24 June 2019.

[32] Pharmacoeconomic Review Report: Edaravone (Radicava): (Mitsubishi Tanabe Pharma Corporation). Ottawa (ON): Canadian Agency for Drugs and Technologies in Health; 2019. 31211530

[33] Fiorentino G, Esquinas AM (2018). Cost-effectiveness associated with amyotrophic lateral sclerosis: some questions and answers pending. Amyotroph Lateral Scler Frontotemporal Degener..

[34] Gruis KL, Chernew ME, Brown DL (2005). The cost-effectiveness of early noninvasive ventilation for ALS patients. BMC Health Serv Res.

[35] Ginsberg G, Lowe S (2002). Cost effectiveness of treatments for amyotrophic lateral sclerosis: a review of the literature. Pharmacoeconomics..

[36] Okano H, Yasuda D, Fujimori K, Morimoto S, Takahashi S (2020). Ropinirole, a new ALS drug candidate developed using iPSCs. Trends Pharmacol Sci.

[37] Cappella M, Ciotti C, Cohen-Tannoudji M, Biferi MG (2019). Gene Therapy for ALS-A Perspective. Int J Mol Sci.

[38] Carpanini SM, Torvell M, Morgan BP (2019). Therapeutic Inhibition of the Complement System in Diseases of the Central Nervous System. Front Immunol.

[39] Statistisches Bundesamt. Gesundheitsausgaben im Jahr 2017. 2019. https://www.destatis.de/DE/Themen/Gesellschaft-Umwelt/Gesundheit/Gesundheitsausgaben/_inhalt.html. Accessed 24 June 2019.

[40] Wetzstein M, Rommel A, Lange C. Informal caregivers - Germany’s largest nursing service. 610 Medizin. Robert-Koch-Institut. GBE kompakt vol. 6 no. 3. 2016. 10.25646/3065.

[41] Chiò A, Gauthier A, Calvo A, Ghiglione P, Mutani R (2005). Caregiver burden and patients' perception of being a burden in ALS. Neurology..

[42] Butterworth P, Pymont C, Rodgers B, Windsor TD, Anstey KJ (2010). Factors that explain the poorer mental health of caregivers: results from a community survey of older Australians. Aust N Z J Psychiatry.

[43] Schorling E, Thiele S, Gumbert L, Krause S, Klug C, Schreiber-Katz O, Reilich P, Nagels K, Walter MC (2019). Cost of illness in Charcot-Marie-tooth neuropathy: results from Germany. Neurology..

[44] Whynes DK (2008). Correspondence between EQ-5D health state classifications and EQ VAS scores. Health Qual Life Outcomes.

[45] Körner S, Kollewe K, Ilsemann J, Müller-Heine A, Dengler R, Krampfl K, Petri S (2013). Prevalence and prognostic impact of comorbidities in amyotrophic lateral sclerosis. Eur J Neurol.

