# Supplementary material for: Costs of illness in amyotrophic lateral sclerosis (ALS): a cross-sectional survey in Germany
Source: Orphanet J Rare Dis. 2020 Jun 12;15:149. doi: 10.1186/s13023-020-01413-9 (PMC7291655; doi:10.1186/s13023-020-01413-9)
Supplement: Supplementary file 1 — Additional file 1. Further details on the utilisation of medical resources. [file 13023_2020_1413_MOESM1_ESM.docx]

**Costs of illness in amyotrophic lateral sclerosis (ALS): a cross-sectional survey in Germany**

**Authors**

Erik Schönfelder^1^, Alma Osmanovic, MD^1^, Lars Hendrik Müschen, MD^1^, Susanne Petri, MD^1^, Olivia Schreiber-Katz, MD^1^

^1^Department of Neurology, Hannover Medical School, Hannover, Germany

| **Additional file 1**  Utilisation of medical resources | | Percent | Absolute number of patients |
| --- | --- | --- | --- |
| Supportive devices (for) | | 80 | 125 |
|  | mobility | 67.9 | 106 |
|  | care | 59 | 92 |
|  | communication | 37.5 | 57 |
|  | activities of daily living | 25 | 39 |
|  | breathing | 30.1 | 47 |
|  | nutrition | 21.2 | 33 |
| Wheelchair use | | 48.1 | 75 |
| Non-invasive ventilation | | 14.7 | 23 |
| Invasive ventilation | | 5.8 | 9 |
| Feeding tube | | 17.3 | 27 |
| Outpatient physician consultation (3 m) | | 84.6 | 132 |
|  | Neurologist | 67.3 | 105 |
|  | General practitioner | 58.3 | 91 |
|  | Domiciliary visit | 25 | 39 |
|  | Respiratory physician | 16 | 25 |
|  | Orthopaedist | 5.8 | 9 |
|  | Gastroenterologist | 2.6 | 4 |
|  | Other | 1.9 | 3 |
| Outpatient hospital consultation (3 m) | | 35.9 | 56 |
|  | Neurologist | 33.3 | 52 |
|  | Respiratory physician | 3.8 | 6 |
|  | Gastroenterologist | 1.9 | 3 |
|  | Orthopaedist | 0.6 | 1 |
|  | Other | 1.9 | 3 |
| Sleep laboratory (6 m) | | 19.2 | 30 |
| Hospitalisation (6 m; reason) (n=57) | | 36.5 | 57 |
|  | ALS diagnosis | 40.4 | 23 |
|  | Ventilator check | 17.5 | 10 |
|  | Supply with feeding tube | 10.5 | 6 |
|  | Pneumonia | 8.8 | 5 |
|  | Dyspnoea | 8.8 | 5 |
| Surgery (ever) | | 21.8 | 34 |
| Inpatient rehabilitation (1 year) | | 23.1 | 36 |
| Further therapies (1 m) | | 96.2 | 150 |
|  | Physiotherapy | 82.7 | 129 |
|  | Speech therapy | 62.2 | 97 |
|  | Ergotherapy | 59.6 | 93 |
|  | Lymph drainage | 12.8 | 20 |
|  | Breathing therapy | 7.7 | 12 |
|  | Nutrition counselling | 1.9 | 3 |
|  | Other | 6.4 | 10 |
| Psychological support (1 m) | | 5.8 | 9 |
| Taking drugs for ALS | | 90.4 | 141 |
|  | Riluzole | 79.5 | 124 |
| Formal care (1 week) | | 41.0 | 64 |
|  | Domestic aid | 31.4 | 49 |
|  | Mobile nursing service | 14.7 | 23 |
|  | (Semi-)Residential care (3 m) | 5.1 | 8 |
|  | Personal assistance | 1.9 | 3 |
|  | Drug delivery service | 1.3 | 2 |
| Legal support (ever) | | 4.5 | 7 |
| House alteration (ever) | | 28.8 | 45 |
| Car alteration (ever) | | 12.8 | 20 |
| Work area alteration (ever) | | 2.6 | 4 |

**Additional file 1. Utilisation of medical resources.** The use of medical resources and demand on informal care (provided by non-professional caregivers) represent the basis for the calculation of direct medical and non-medical costs. The recall periods can be found in brackets, if not we assessed the present status. Patients were asked only to give statements strictly related to their ALS diagnosis, so that the recall period “ever” means since disease onset. Abbreviations: m = months, n = number, ALS = amyotrophic lateral sclerosis.
