# Supplementary material for: Costs of illness in amyotrophic lateral sclerosis (ALS): a cross-sectional survey in Germany
Source: Orphanet J Rare Dis. 2020 Jun 12;15:149. doi: 10.1186/s13023-020-01413-9 (PMC7291655; doi:10.1186/s13023-020-01413-9)
Supplement: Supplementary file 2 — Additional file 2. Detailed description of cost estimation methodology. [file 13023_2020_1413_MOESM2_ESM.docx]

**Costs of illness in amyotrophic lateral sclerosis (ALS): a cross-sectional survey in Germany**

**Authors**

Erik Schönfelder^1^, Alma Osmanovic, MD^1^, Lars Hendrik Müschen, MD^1^, Susanne Petri, MD^1^, Olivia Schreiber-Katz, MD^1^

^1^Department of Neurology, Hannover Medical School, Hannover, Germany

**Additional file 2**

**Methods**

**Detailed description of cost estimation methodology**

Briefly summarized, the detailed cost estimation of the different cost factors was performed as follows. For outpatient physician consultations, costs per patient-doctor contact were calculated based on total specialty-specific honoraria in 2018 divided through the number of patient contacts per year [1]. We also took price-increase rates [2, 3], expenditures of private health insurances and costs of laboratory medicine into account. Inpatient hospital treatments were calculated based on the individual duration and daily rates for normal care and intensive care units as recommended [1]. For outpatient hospital consultations in specialized neuromuscular outpatient clinics we used our own in-house remuneration. In our patient cohort, rehabilitation exclusively took place in an inpatient setting so that costs per day were used as calculation basis. The German “Heilmittelkatalog” [4] gives details about adequate further therapies for each disease, inter alia physiotherapy, ergotherapy, nutritional and speech therapy. We then used the remuneration agreements between care providers and health insurances to define the costs per unit, depending on whether the patient had a statutory or private health insurance [5]. Moreover, patients were asked to provide information on currently used medical devices and for how long they have been using them. To calculate monthly costs for these supportive devices we considered patients’ expenses or, if these were unknown, roughly approximated prices as suggested [1]. Hereby, we assumed that the purchase of the medical aid is only necessary once during three years, the median time span of the course of amyotrophic lateral sclerosis (ALS) [6]. Drug costs were monetarily evaluated based on public prices for Germany depending on dosage and package size [7].

Care is differentiated into formal (professional) and informal care which is provided by non-trained personnel, mainly family members. We subdivided professional care into residential care, which is reimbursed per day in Germany [8], mobile nursing service for which we calculated the average basic care prices per hour of nearby mobile nursing services [9] and domestic aid for which we assumed the statutory minimum wage in Germany [10]. To calculate informal care costs, different methods do exist. In this study, we replaced the time of care provided by informal caregivers by the statutory minimum wage for the caring sector in order to estimate the costs that would have risen if the care had been provided by professional caregivers instead [1].

For the estimation of indirect costs, we used the human capital approach. Actual wages of patients and caregivers led to their ALS-related loss of income due to reduction of working time, absent days and early retirement (below the age of 67 years) [5]. All costs were extrapolated to one year while assuming a stable status over this time period.

**References**

1. Bock JO, Brettschneider C, Seidl H, Bowles D, Holle R, Greiner W, König HH. Standardisierte Bewertungssätze aus gesellschaftlicher Perspektive für die gesundheitsökonomische Evaluation. Baden-Baden: Nomos Verlagsgesellschaft; 2015.

2. Kassenärztliche Bundesvereinigung. Kennzahlen der Abrechnungsgruppen 1. Quartal 2009 bis 4. Quartal 2016. 2018. https://www.kbv.de/media/sp/20180801_Honorarbericht_2016Q4_Tabellen_AGP.xlsx. Accessed 15 April 2019

3. Kassenärztliche Bundesvereinigung. Kennzahlen der Abrechnungsgruppen 1. Quartal 2013 bis 1. Quartal 2018. 2019. https://www.kbv.de/media/sp/Honorarbericht_Tabellen.xlsx. Accessed 15 April 2019

4. IntelliMed GmbH. Der Heilmittelkatalog. 2017. https://heilmittelkatalog.de/files/luxe/hmkonline/online/index.htm. Accessed 31 January 2019

5. Krauth C, Hessel F, Hansmeier T, Wasem J, Seitz R, Schweikert B. Empirische Bewertungssätze in der gesundheitsökonomischen Evaluation -- ein Vorschlag der AG Methoden der gesundheitsökonomischen Evaluation (AG MEG) [Empirical standard costs for health economic evaluation in Germany -- a proposal by the working group methods in health economic evaluation]. Gesundheitswesen. 2005;67(10):736–746. doi:10.1055/s-2005-858698

6. Roche JC, Rojas-Garcia R, Scott KM, Scotton W, Ellis CE, Burman R, Wijesekera L, Turner MR, Leigh PN, Shaw CE, Al-Chalabi A. A proposed staging system for amyotrophic lateral sclerosis. Brain. 2012;135(3):847-852. https://doi.org/10.1093/brain/awr351

7. Rote Liste® Service GmbH. Rote Liste. 2019. https://www.rote-liste.de. Accessed 30 January 2019

8. Bundesgesundheitsministerium. Sechster Bericht der Bundesregierung über die Entwicklung der Pflegeversicherung und den Stand der pflegerischen Versorgung in der Bundesrepublik Deutschland. 2016. https://www.bundesgesundheitsministerium.de/fileadmin/Dateien/5_Publikationen/Pflege/Berichte/6.Pflegebericht.pdf. Accessed 25 March 2019

9. Verband der Ersatzkassen e. V. (vdek) Pflegelotse. https://www.pflegelotse.de/presentation/pl_startseite.aspx. Accessed 25 March 19

10. Statistisches Bundesamt. Arbeitsmarkt und Verdienste; Auszug aus dem Datenreport 2018. 2018. https://www.destatis.de/DE/Service/Statistik-Campus/Datenreport/Downloads/datenreport-2018-kap-5.pdf?__blob=publicationFile. Accessed 25 June 2019
